# Helicobacter pylori infection increases the risk of dyslipidemia in Chinese diabetic Population: a retrospective cross-sectional study

**DOI:** 10.1186/s12879-024-09597-2

**Published:** 2024-07-25

**Authors:** Chaoyu Yang, Ningning You, Yi Chen, Jinshun Zhang

**Affiliations:** 1 Department of Gastroenterology, Taizhou Hospital of Zhejiang Province affiliated to Wenzhou Medical University, Taizhou, 317000 Zhejiang China; 2 Health Management Center, Taizhou Hospital of Zhejiang Province affiliated to Wenzhou Medical University, Taizhou, 317000 Zhejiang China

**Keywords:** *Helicobacter pylori*, Dyslipidemia, Diabetes mellitus

## Abstract

**Background:**

In contemporary times, increased prevalence of *Helicobacter pylori* (*H. pylori*) infection and elevated dyslipidemia levels present substantial public health challenges. However, the relationship between *H. pylori* and dyslipidemia remains inconclusive. No studies have yet conducted a population-based classification to investigate the impact of *H. pylori* infection on dyslipidemia in individuals with diabetes.

**Methods:**

A retrospective cohort study was carried out on a total of 60,535 individuals who underwent health check-ups at the Health Examination Center in Taizhou Hospital from 2017 to 2022. Physical measurements, hematological markers and detection of *H. pylori* were gathered from all patients. The study population was further stratified into diabetic and non-diabetic groups for analysis.

**Results:**

*H. pylori* infection was found to be an autonomous risk factor for dyslipidemia based on the results of multivariate logistic regression analysis (OR = 1.13, 95% CI: 1.03–1.24). However, no notable effect on dyslipidemia in the non-diabetic group was observed. Furthermore, at the follow-up, the group with persistent negative showed a significantly lower incidence ratio of dyslipidemia compared to the group with persistent infection (*P* = 0.006). The persistent negative group exhibited a significantly higher rate of improvement in dyslipidemia compared to the new infection group (*P* = 0.038).

**Conclusions:**

In the diabetic population, the presence of *H. pylori* infection heightens the propensity for developing dyslipidemia. Therefore, the implementation of efficient eradication strategies for *H. pylori* infection could potentially lead to a decrease in the occurrence of dyslipidemia among individuals with diabetes.

## Introduction

As one of the most pervasive chronic infectious diseases in humans, *Helicobacter pylori* (*H. pylori*) colonizes the gastric mucosa in around half of all adults [1–5]. The majority of infections are acquired during early childhood and persist throughout an individual’s lifetime [4–6]. *H. pylori* not only serves as a primary contributor to clinical gastrointestinal disorders, such as stomach or duodenal ulcers and gastric cancer [3, 4, 7], but it is also linked to various extragastric conditions. Several independent studies have revealed associations between *H. pylori* infection and cardiovascular disease (CVD), type 2 diabetes mellitus (T2DM), as well as metabolic syndrome [8, 9].

Dyslipidemia is a well-established independent risk factor for CVD, stroke, and atherosclerosis [10], characterized by increased concentrations of total triglycerides (TG), cholesterol (TC), low-density lipoprotein cholesterol cholesterol (LDL-c), or reduced high-density lipoprotein cholesterol (HDL-c) [11, 12]. In china, the prevalence of dyslipidemia exhibited a rapid increase from 2002 to 2015, while it is projected that annual CVD incidents will experience a surge of over 50% between 2010 and 2030 [12]. Recently, numerous studies have unveiled the impact of dyslipidemia on the human microbiome. The microbiome plays an essential role in lipid metabolism within the human body [13–15], as it constitutes a complex and metabolically active ecosystem that is crucial for processing dietary components [15]. A retrospective study, which included 5,077 Japanese patients, revealed a profound correlation between *H. pylori* infection and elevated LDL-c levels as well as reduced HDL-c levels among male patients [16]. Similarly, another Korean cross-sectional analysis involving 37,263 participants showed the influence of *H. pylori* infection on dyslipidemia, including increased TC and LDL-c, and decreased HDL-c [17]. In contrast to these findings, a study conducted on 58 patients showed that no notable alterations were observed in TC, TG, HDL-c or LDL-c levels subsequent to the eradication of *H. pylori* [18]. Meanwhile, a meta-analysis did not find any significant correlation between *H. pylori* seropositivity and TC or TG levels [19]. Thus, this ongoing controversy highlights the relationship. In addition, a systematic review study has unveiled the correlation between *H. pylori* infection and homeostasis model assessment IR (HOMA-IR), an indicator of insulin resistance (IR) which plays a crucial role in the pathogenesis of T2DM [20]. Diabetes and dyslipidemia are interrelated metabolic disorders that reciprocally influence each other. However, no studies have been undertaken to explore the impact of *H. pylori* infection on dyslipidemia in individuals with diabetes as yet.

In this study, a retrospective cross-sectional survey and cohort investigation were carried out to elucidate the potential contribution of *H. pylori* infection to the risk of dyslipidemia in the Chinese diabetic population. Furthermore, a comprehensive analysis was performed to investigate the correlation between *H. pylori* infection and dyslipidemia in individuals with T2DM.

## Methods

### Sample population

Between January 2017 and September 2022, a cumulative number of 60,535 patients aged over 20 years underwent extensive health screening assessments at the Health Management Center of Taizhou Hospital in Zhejiang Province, China. The exclusions for invalid and missing data are as follows: (1) age below 18 years or above 80 years; (2) lack of comprehensive clinical data and personal history; (3) a history of malignancy, abnormal liver or kidney function, thyroid disease and severe obesity; (4) previous or current *H. pylori* eradication therapy and dyslipidemia therapy [21]. Finally, 46,808 participants underwent a single physical examination, while 24,731 completed multiple physical examinations. Data from the initial examination were utilized for the cross-sectional analysis among individuals who underwent multiple examinations, as well as those who had only undergone one examination. For the cohort analysis, we specifically selected data from the initial and final health checkups with an interval time more than 12 months. This study has been granted ethical approval by the ethics committee at Taizhou Hospital (K20220790).

### Clinical data collection

The proficient nurses at the Health center gathered and documented basic information, including age, gender, smoking habits, alcohol consumption, and personal medical history of the patients. Additionally, they also measured diastolic blood pressure (DBP) and systolic blood pressure (SBP) after a 5-minute period of rest in a calm seated position. After a 12-hour overnight fast, venous blood samples were collected from individuals in the morning on an empty stomach to measure laboratory serum biochemical parameters, including TG, TC, HDL-c, LDL-c, uric acid, creatinine and glycated hemoglobin A1c (HbA1c). fasting blood glucose (FBG) was collected after an 8-hour fast. The calculation formula of BMI index was [weight/height squared (kg/m^2^)].

### Definition of dyslipidemia and *H. pylori* infection

Dyslipidemia was defined as meeting any of the following criteria: TC levels ≥ 240 mg/dL, TG levels > 200 mg/dL, reduced HDL-c levels < 40 mg/dL for men and < 50 mg/dL for women, or elevated LDL-c levels ≥ 130 mg/dL. The occurrence of dyslipidemia was determined based on the manifestation of either “borderline high LDL-c” or “low HDL-c,” whichever occurred first [11]. The methodology used for detecting *H. pylori* was the ^13^C/^14^C-urea breath test (^13^C/^14^C-UBT). The specific steps of the ^13^C-UBT were as follows: (1). Collect the initial breath sample after fasting for 3 h; (2). Take a ^13^C urea capsule and wait for 30 min; (3). Exhale into the special gas colloection bag again; (4). Analyze the results on the instrument. The specific steps of the ^14^C-UBT were as follows: (1). Swallow a ^14^C urea capsule with warm water; (2). Wait for 15 min; (3). Continuously exhale into the gas collection bag for 1–3 min; (4). Insert the bag into the detector for analysis [22].

### Definition of *H. Pylori* status

Studied participants were classified into four groups: persistent infection group ( *H. pylori* status at the baseline and endpoint positive), eradication infection group (*H. pylori* positive at the first visit, with *H. pylori* negative at the endpoint), persistent negative group (baseline and endpoint *H. pylori* status negative) and New infection group (*H. pylori* negative at the baseline, with *H. pylori* positive at the endpoint). Endpoints were defined as the time of the second or more physical examinations in which dyslipidemia was developed and, if dyslipidemia was not developed persistently, the time of the latest follow up between January, 2017 and September, 2022.

### Statistical analysis

In this study, continuous and categorical variables were presented as mean ± standarddeviation (SD) and counts or percentages, using Student’s t test or one-way ANOVA and chi-square test for comparisons between the risk groups and the subgroups, respectively. Multivariate logistic regression models were used to estimate odds ratios (ORs) with 95% confidence intervals (CIs) for dyslipidemia associated with *H. pylori* infection, while controlling for potential confounding factors. Four adjustment models were fitted for other risk factors. All statistical analyses were performed using R version 4.1.3, and variables with a *P*-value less than 0.05 were considered statistically significant.

## Results

### Part I

#### Baseline demographic characteristics

The baseline characteristics of all enrolled participants are shown in Table 1. Out of a total of 60,535 individuals who underwent physical examinations, 37,286 (61.6%) were male and 23,249 (38.4%) were female. The infection rate of *H. pylori* was 37.0%. Among those who are positive for the *H. pylor*i infection, there is a higher proportion of males, 62.7% vs. 60.9%. Compared to the negative group, individuals with diabetes had a higher rate of *H. pylori* infection, 15.1% vs. 14.4% (*P* = 0.02). However, there was no statistical difference between dyslipidemia and *H. pylori* infection, 37.0% vs. 36.2% (*P* = 0.069).

**Table 1:** Baseline characteristics of the entire physical examination cohorts

Variable	H. pylori-negative (*n* = 38,119)	H. pylori-positive (*n* = 22,416)	*P* value
Gender (n, %)			< 0.001
Male	23,224(60.9%)	14,062(62.7%)	
Age (years)	49.3 ± 12.5	49.8 ± 12.5	< 0.001
Fasting blood glucose (mmol/L)	5.50 ± 1.5	5.57 ± 1.66	< 0.001
Glycated hemoglobin A1c (%)	5.91 ± 0.93	5.96 ± 1.02	< 0.001
Triglycerides (mmol/L)	2.00 ± 1.81	1.96 ± 1.80	0.017
Total cholesterol (mmol/L)	5.14 ± 1.03	5.11 ± 1.01	< 0.001
Low density lipoprotein (mmol/L)	2.80 ± 0.77	2.77 ± 0.75	< 0.001
High density lipoprotein (mmol/L)	1.42 ± 0.33	1.30 ± 0.32	< 0.001
Uric Acid (umol/L)	344.85 ± 91.1	346.19 ± 92.9	> 0.05
Creatinine (umol/L)	70.3 ± 19.7	71.2 ± 21.8	< 0.001
Mean BMI (kg/m²)	24.4 ± 3.3	24.6 ± 3.7	< 0.001
Systolic blood pressure (mmHg)	127.2 ± 18.0	128.1 ± 18.8	< 0.001
Diastolic pressure (mmHg)	76.2 ± 11.9	76.7 ± 12.1	< 0.001
Dyslipidemia (n, %)	13,806(36.2%)	8284(37.0%)	> 0.05
Diabetes (n, %)	5497(14.4%)	3389(15.1%)	0.02
Smoking (n, %)	7807(20.5%)	4727(21.1%)	> 0.05
Alcohol consumption (n, %)	5535(14.5%)	3375(15.1%)	> 0.05

**Fig. 1:**
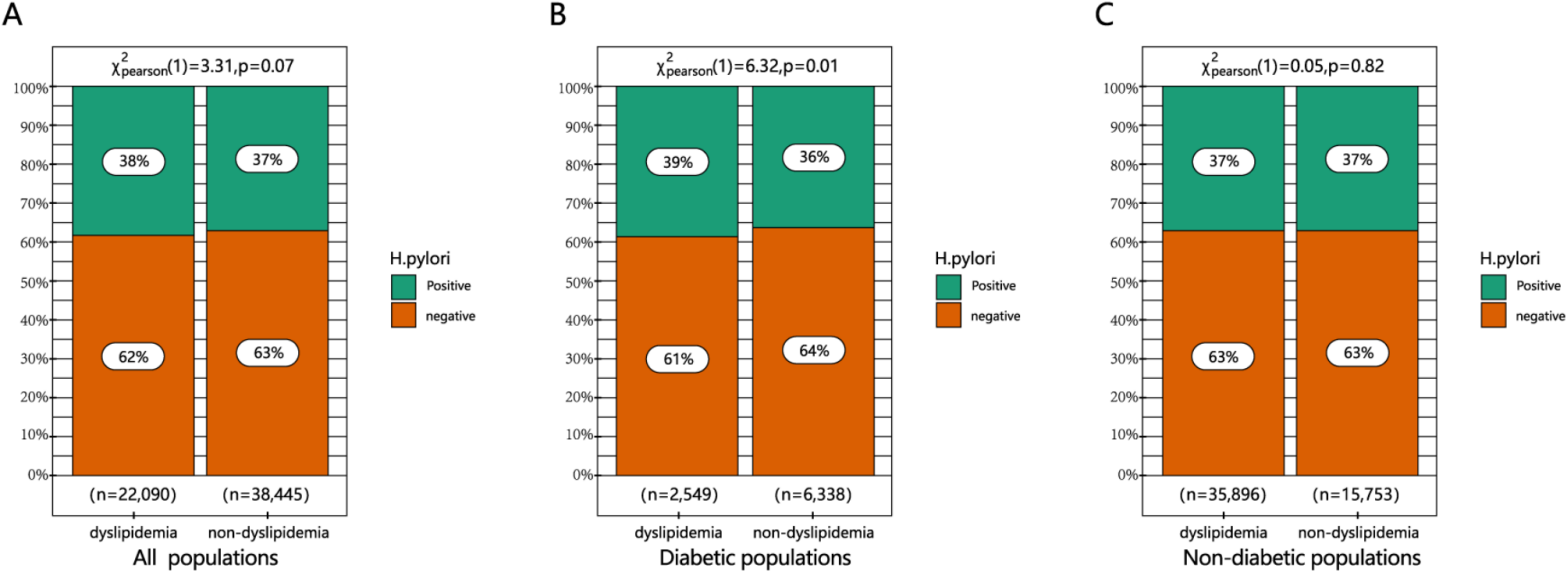
Relationship between *Helicobacter pylori* infection and dyslipidemia in diverse populations, (**A**) All populations. (**B**) Diabetic populations. (**C**) Non-diabetic populations

To further explore the potential link between *H. pylori* infection and the development of dyslipidemia in diverse populations, a subgroup analysis was performed using univariate analysis to examine the risk factors influencing dyslipidemia in both diabetic and non-diabetic individuals. The basic characteristics of dyslipidemia patients in the non-diabetic and diabetic groups are presented in Table 2. In the non-diabetic group, individuals with dyslipidemia exhibited a significant elevation in TG levels (2.35 ± 2.02 vs. 1.05 ± 0.32) and LDL-c levels (2.99 ± 0.76 vs. 2.29 ± 0.43). However, there was no significant correlation between *H. pylori* infection and dyslipidemia (*P* = 0.82) among the non-diabetic group. Moreover, significant statistical differences were observed in age, gender, FBG, BP, lipid parameters, and *H. pylori* infection (*P* = 0.012) between dyslipidemia subjects within the diabetic group and non-dyslipidemia subjects. The heterogeneity in *H. pylori* infection among diverse populations of patients with dyslipidemia is depicted in Fig. 1.

**Table 2:** Baseline characteristics of dyslipidemia population in the non-diabetic and diabetic group

Variable	Non-diabetic group			Diabetic group		
	Non-Dyslipidemia (*n* = 15,753)	Dyslipidemia (*n* = 35,896)	*P* value	Non-Dyslipidemia (*n* = 2549)	Dyslipidemia (*n* = 6337)	*P* value
Gender (n, %)			< 0.001			< 0.001
Male	9698(61.6%)	21,284(59.3%)		1967(77.2%)	4337(68.4%)	
Age (years)	46.7 ± 12.8	48.75 ± 11.95	< 0.001	59.1 ± 11.1	56.8 ± 10.9	< 0.001
Fasting blood glucose (mmol/L)	5.02 ± 0.54	5.12 ± 0.56	< 0.001	7.57 ± 2.43	8.22 ± 2.81	< 0.001
Glycated hemoglobin A1c (%)	5.56 ± 0.35	5.67 ± 0.35	< 0.001	7.42 ± 1.33	7.7 ± 1.57	< 0.001
Triglycerides (mmol/L)	1.05 ± 0.32	2.35 ± 2.02	< 0.001	1.06 ± 0.33	2.32 ± 1.95	< 0.001
Total cholesterol (mmol/L)	4.46 ± 0.5	5.39 ± 1.02	< 0.001	4.58 ± 0.71	5.54 ± 1.22	< 0.001
Low density lipoprotein (mmol/L)	2.29 ± 0.43	2.99 ± 0.76	< 0.001	2.37 ± 0.54	3.05 ± 0.85	< 0.001
High density lipoprotein (mmol/L)	1.45 ± 0.26	1.42 ± 0.35	< 0.001	1.39 ± 0.26	1.32 ± 0.33	< 0.001
Uric Acid (umol/L)	335.86 ± 88.41	350.06 ± 92.41	< 0.001	341.75 ± 85.08	348.83 ± 87.81	< 0.001
Creatinine (umol/L)	70.46 ± 23.40	70.5 ± 17.48	> 0.05	72.77 ± 19.63	70.62 ± 27.80	< 0.001
Mean BMI (kg/m²)	23.71 ± 3.18	24.50 ± 3.23	< 0.001	25.43 ± 3.18	26.03 ± 3.39	< 0.001
Systolic blood pressure (mmHg)	124.04 ± 17.52	126.87 ± 17.86	< 0.001	136.67 ± 18.96	136.81 ± 18.81	< 0.001
Diastolic blood pressure (mmHg)	74.51 ± 11.80	76.39 ± 11.90	< 0.001	79.17 ± 11.44	80.25 ± 11.89	> 0.05
*H. pylori* (n, %)	5815(36.9%)	13,212(36.8%)	> 0.05	920(36.1%)	2469(39.0%)	0.012
Smoking (n, %)	2880(18.3%)	7096(19.8%)	< 0.001	1819(71.4%)	739(11.7%)	> 0.05
Alcohol consumption (n, %)	1984(12.6%)	5070(14.1%)	< 0.001	1198(47.0%)	479(7.6%)	> 0.05

**Fig. 2:**
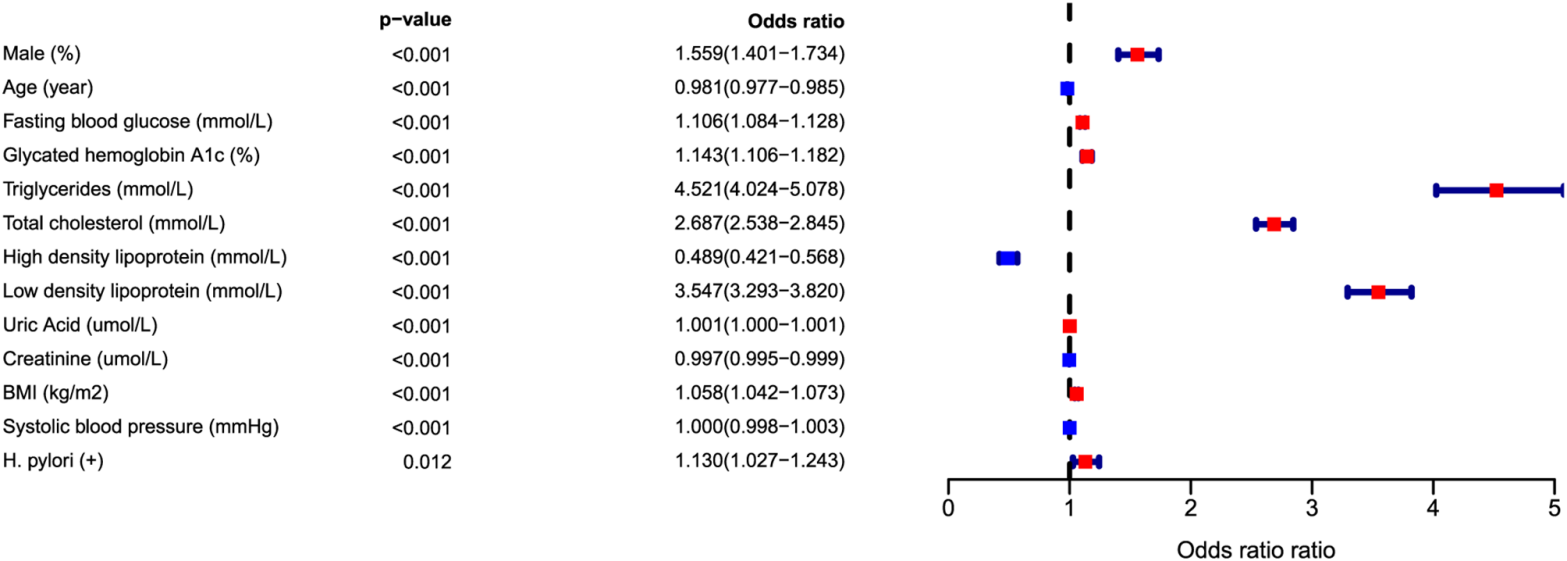
Univariate analysis of dyslipidemia risk factors in diabetes population

In the diabetic group, univariate logistic regression models showed that *H. pylori* infection was associated with a higher risk of dyslipidemia (OR = 1.13, 95%CI: 1.03–1.24), as illustrated in Fig. 2. Furthermore, gender, age, hypertension, BMI, and HbA1c were identified as independent risk factors for dyslipidemia (*P* < 0.05). Four models with progressive degrees of adjustment were fitted for potential confounders. In addition to gender and age, adjustments were made for hypertension, HbA1c, smoking, and alcohol consumption. The presence of *H. pylori* infection remains a risk factor for dyslipidemia, as shown in Table 3.

**Table 3:** Relationship between *H. Pylori* infection and dyslipidemia in different regression models

	OR (95%CI)	*p* value
Modle1	1.14(1.04–1.25)	0.008
Modle2	1.12(1.01–1.24)	0.031
Modle3	1.11(1.01–1.23)	0.031
Modle4	1.14(1.04–1.26)	0.006
Model 1 is adjusted for age, gender.		
Model 2 is adjusted for age, gender, hypertension.		
Model 3 is adjusted for age, gender, Glycated hemoglobin A1c .		
Model 4 is adjusted for age, gender, smoking, alcoholic consumption.		

### Part I

#### Correlation between *H. Pylori* infection status and dyslipidemia

**Fig. 3:**
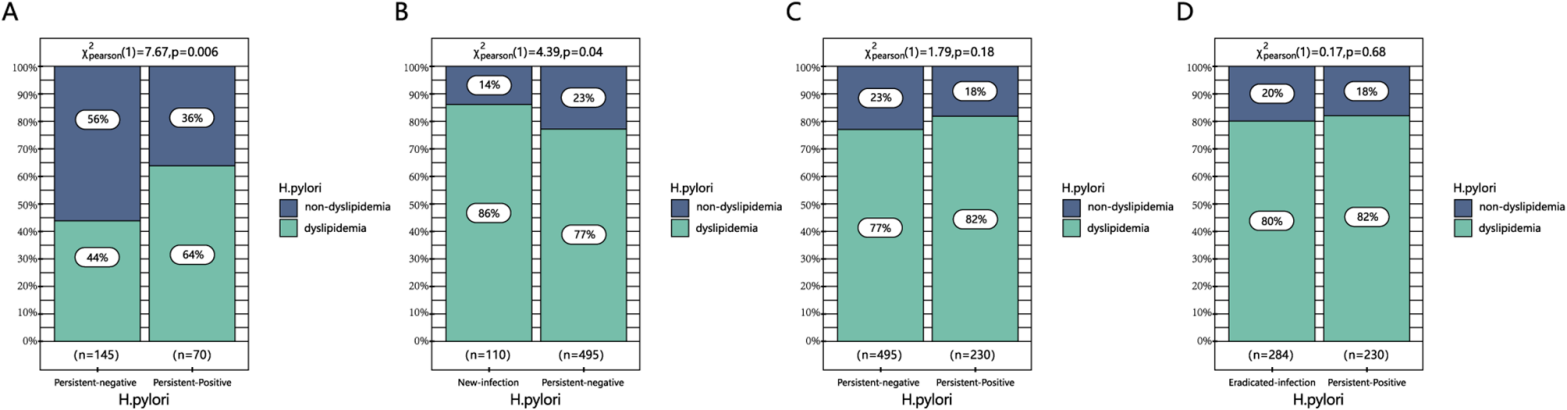
Effect of different *Helicobacter pylori* infection statuses on the development of dyslipidemia: (**A**) Comparison between the persistent infection group and the persistent negative group. Effect of different Helicobacter pylori infection statuses on the improvement of dyslipidemia: (**B**) Comparison between the persistent negative group and the new infection group. (**C**) Comparison between the persistent negative group and the persistent infection group. (**D**) Comparison between the eradicated infection group and the persistent infection group

Among the 1633 patients with diabetes, a total of 1477 individuals underwent follow-up for more than one year. Out of these, 358 (24.2%) patients did not have dyslipidemia during their initial physical examination. The incidence rates of dyslipidemia were 44.1% and 64.3% in the persistent negative and positive groups, respectively. Additionally, the eradicated infection group had a rate of 69.6%, while the new infection group had a rate of 67.7%. The persistent negative group exhibited a significantly lower incidence ratio of dyslipidemia compared to that in the persistent infection group (*P* = 0.006), as depicted in Fig. 3A.

Out of the 1,477 patients with diabetes, 1,119 (75.8%) individuals had dyslipidemia during their first visit. The improvement rates for dyslipidemia were 22.6%, 18.3%, 19.7%, and 13.6% in the persistent negative, persistent positive, eradicated infection, and new infection groups respectively. The new infection group exhibited a significantly diminished rate of improvement in dyslipidemia compared to the persistently negative group (*P* = 0.038). In addition, the rate in the persistent positive group was found to be lower than that observed in both the persistent negative and eradication groups; however, this difference did not reach statistical significance, as shown in Fig. 3B, C, D.

## Discussion

The correlation between *H. pylori* infection and dyslipidemia remains a topic of controversy due to the lack of studies that stratify populations based on diabetes status, which is necessary for accounting potential confounding effects when assessing the impact of *H. pylori* infection on dyslipidemia. The association between *H. pylori* infection and dyslipidemia has been validated in this study, which was conducted on a large sample population. Nevertheless, considering the distinctive characteristics of IR and hereditary vulnerability in diabetic individuals compared to non-diabetic individuals, it is crucial to investigate the variations in the impact of *H. pylori* infection on dyslipidemia.

This large cross-sectional research has undertaken a separate analysis of the association between *H. pylori* infection and dyslipidemia, with a specific focus on patients with diabetes mellitus. The prevalence of *H. pylori* infection was found to be higher in the dyslipidemia group compared to the non-dyslipidemia group, indicating a positive correlation between *H. pylori* infection and dyslipidemia. Furthermore, even after adjusting for multiple confounding variables, *H. pylori* infection remains a significant independent risk factor for the progression of dyslipidemia. At the same time, we observed that dyslipidemia was more common in females and populations with higher levels of FBG, uric acid, or BMI. Indeed, these aforementioned factors were also independent risk factors for dyslipidemia. This means that these people are vulnerable to *H. pylori* infection. In addition, the further cohort study revealed a significantly higher incidence of dyslipidemia in the persistent infection group compared to the persistent negative group, while the rate of improvement in dyslipidemia was notably lower in the new infection group than that observed in the persistent negative group. These pieces of evidence suggested that *H. pylori* infection may potentially contribute to the occurrence and progression of dyslipidemia. It was consistent with a large cohort study conducted by Park, Y., et al [11]. This implies that eradicating *H. pylori* infection within diabetic individuals may have favorable implications in terms of mitigating the incidence of dyslipidemia. Therefore, internists should carefully consider the increased risk of dyslipidemia in patients with *H. pylori* and advocate for eradication therapy, especially in individuals with diabetes.

The pathogenesis of dyslipidemia can be ascribed to a blend of hereditary, ecological, and metabolic elements [23]. Several studies have indicated a potential correlation between dyslipidemia and *H. pylori* infection, further emphasizing the multifactorial nature of this condition [11, 24]. Recent evidence has demonstrated that *H. pylori* infection induces the upregulation of various inflammatory mediators, including macrophages, tumor necrosis factors (TNF), and several interleukins. The release of macrophages and interleukins results in the accumulation of active substances, exacerbating oxidative stress reactions in the body and promoting accelerated mobilization of fat [25]. TNF-α impedes the activity of lipoprotein lipase, facilitating lipid mobilization in tissues and consequently inducing perturbations in lipid and lipoprotein metabolism [26–28]. Additionally, Other studies have shown that diabetic patients may have a higher risk of developing Non-alcoholic fatty liver disease (NAFLD) due to *H. pylori* infection, and there is a non-linear relationship between *H. pylori* infection and HbA1c. This could be attributed to the fact that both dyslipidemia and NAFLD are metabolic-related diseases, and *H. pylori* infection leads to increased IR as well as changes in lipid and lipoprotein metabolism [29–32]. The infection may worsen damage to pancreatic cells, aggravating insufficient insulin secretion and leading to abnormal hormone secretion in mucosal inflammation [33–35]. This could potentially explain the increased occurrence of dyslipidemia among *H. pylori*-positive individuals within the diabetic population as observed in this study.

The strengths of this research lie in the implementation of a cross-sectional design, a comparatively large sample size, population categorization, and the inclusion of a cohort to evaluate the impact of current infection and its eradication on lipid profiles, which enables us to determine the causal relationship accurately. The outcomes of this study have confirmed a positive correlation between dyslipidemia and *H. pylori* infection in diabetic patients, and persistent *H. pylori* infection increases the risk of developing dyslipidemia. To our knowledge, this study represents a pioneering investigation into the potential impact of *H. pylori* infection on dyslipidemia within a substantial diabetic population. Nonetheless, it is imperative to acknowledge that certain limitations were also evident in this study. First of all, the inherent constraints of retrospective research design should be recognized. Second, this study is limited to a single center, which implies certain constraints. To establish more reliable evidence, it may be necessary to conduct multicenter and longitudinal studies. Third, this study focuses on diabetic populations who have undergone regular health check-ups; therefore, caution should be exercised when extrapolating these findings to broader populations. In addition, the *H. pylori* infection status was exclusively determined through the ^13^C/^14^C-UBT, without resorting to gastroscopy biopsy or microbial culture for diagnosing *H. pylori* infection. Since other urease-producing bacteria were found in the stomach, this could lead to a false positive result for *H. pylori* infection in the urea test. There are many alternative non-invasive diagnostic techniques available for diagnosing *H. pylori* infection, such as serological testing for anti-*H. pylori* antibodies, the stool antigen test (SAT), and the direct detection of *H. pylori* genetic material in stool via PCR. Nevertheless, previous studies have shown a robust concordance between the ^13^C/^14^C-UBT and gastroscopy biopsy [36], implying that the ^13^C/^14^C-UBT displays exceptional diagnostic accuracy and commendable performance [3, 37, 38]. Finally, despite the comprehensive inclusion of significant confounding variables in the multivariable analysis, the potential existence of residual confounders due to measurement errors or insufficient measurements that cannot be entirely ruled out.

## Conclusion

*H. pylori* infection is identified as an autonomous risk factor for dyslipidemia in the diabetic population. Furthermore, prolonged *H. pylori* infection may augment the likelihood of developing dyslipidemia. Consequently, eradicating *H. pylori* infection among individuals with diabetes could potentially contribute to a reduction in the incidence of dyslipidemia.

## Data Availability

The dataset utilized in the present study can be obtained from the corresponding author upon a reasonable request.

## Abbreviations

H. pylori: Helicobacter pylori
CVD: Cardiovascular disease
T2DM: Type 2 diabetes mellitus
TG: Total triglycerides
TC: Total cholesterol
HDL-c: High-density lipoprotein cholesterol
LDL-c: Low-density lipoprotein cholesterol
IR: Indicator of insulin resistance
HbA1c glycated: Hemoglobin A1c
TNF: Tumor necrosis factor
^13^C/^14^C-UBT: ^13^C/^14^C-urea breath test
SAT: Stool antigen test
NAFLD: Non-alcoholic fatty liver disease
